# Tree Branching: Leonardo da Vinci's Rule versus Biomechanical Models

**DOI:** 10.1371/journal.pone.0093535

**Published:** 2014-04-08

**Authors:** Ryoko Minamino, Masaki Tateno

**Affiliations:** Nikko Botanical Garden, Graduate School of Science, The University of Tokyo, Nikko, Tochigi, Japan; Mayo Clinic College of Medicine, United States of America

## Abstract

This study examined Leonardo da Vinci's rule (*i.e.*, the sum of the cross-sectional area of all tree branches above a branching point at any height is equal to the cross-sectional area of the trunk or the branch immediately below the branching point) using simulations based on two biomechanical models: the uniform stress and elastic similarity models. Model calculations of the daughter/mother ratio (*i.e.*, the ratio of the total cross-sectional area of the daughter branches to the cross-sectional area of the mother branch at the branching point) showed that both biomechanical models agreed with da Vinci's rule when the branching angles of daughter branches and the weights of lateral daughter branches were small; however, the models deviated from da Vinci's rule as the weights and/or the branching angles of lateral daughter branches increased. The calculated values of the two models were largely similar but differed in some ways. Field measurements of *Fagus crenata* and *Abies homolepis* also fit this trend, wherein models deviated from da Vinci's rule with increasing relative weights of lateral daughter branches. However, this deviation was small for a branching pattern in nature, where empirical measurements were taken under realistic measurement conditions; thus, da Vinci's rule did not critically contradict the biomechanical models in the case of real branching patterns, though the model calculations described the contradiction between da Vinci's rule and the biomechanical models. The field data for *Fagus crenata* fit the uniform stress model best, indicating that stress uniformity is the key constraint of branch morphology in *Fagus crenata* rather than elastic similarity or da Vinci's rule. On the other hand, mechanical constraints are not necessarily significant in the morphology of *Abies homolepis* branches, depending on the number of daughter branches. Rather, these branches were often in agreement with da Vinci's rule.

## Introduction

Many studies have examined tree design, which has led to several empirical rules. Leonardo da Vinci proposed that the sum of the cross-sectional area of all tree branches above a branching point at any height is equal to the cross-sectional area of the trunk or the branch immediately below the branching point [1]. This relationship can also be expressed by stating that the branch cross-sectional area below a given branching node is equal to the sum of the cross-sectional areas of daughter branches above the node [2]–[6]. This is known as Leonardo da Vinci's rule, or the area-preserving rule [2]. However, not all branches correspond to da Vinci's rule. Sone et al. [4] found that the average yearly growth of the cross-sectional area of a branch was less than the sum of growth of its daughter branches. This is because the proportion of the current-year growth area to the cross-sectional area of the branch is almost always greater in small, young branches than in large, old branches. The authors noted that da Vinci's rule would not hold if the decrease in basipetal growth was repeated every year. Sone et al. [6] demonstrated that in *Acer rufinerve*, only branches that have experienced shedding follow da Vinci's rule.

One reason for the lack of agreement with da Vinci's rule in some branches may be expressed in terms of their biomechanical structure. For trees, safeguarding against mechanical stress such as gravity, during morphogenesis, is essential for survival [7]–[9]. The magnitude of the impact of mechanical stress varies greatly with tree form. Therefore, the prevailing view has been that a branch adjusts itself to its mechanical constraints, and this has led to attempts to analyze the biomechanical designs of trees. Indeed, variation in the mechanical environment does have an effect on tree morphology. Trees that have been tethered in place with ropes to reduce mechanical stimulation by wind forces grew taller than control specimens [10]. Furthermore, the safety factor, *i.e.*, the ratio between critical buckling height (which is estimated from the trunk diameter) and actual tree height, is small when the mechanical safety of the tree is low; safety factors of trees growing in protected conditions within dense forests are lower than those of trees growing in open environments where they are exposed to stronger wind forces [11]. These relationships suggest that (i) there is a biomechanical limitation that changes the relationship between the diameter and length of a trunk or branch (a reflection of mechanical safety), and (ii) the magnitude of branch biomechanical safety should be given a proper value. Hydraulic resistance in a tree also affects its height growth [12]. Taneda and Tateno [13] compared mechanical and hydraulic limitations and concluded that the partitioning of biomass in current shoots of both angiosperms and gymnosperms is governed mainly by the mechanical support criterion (although under some circumstances, gymnosperms may be more affected by the water transport criterion).

The present study focuses on the validity of da Vinci's rule in the context of mechanical limitation and two relevant biomechanical hypotheses: the uniform stress hypothesis and the elastic similarity hypothesis [8], [14]–[16]. In the uniform stress hypothesis, the mechanical safety of a branch, or the mechanical stress that acts on a branch, is assumed to be uniform at any point along the branch. In previous studies, it has been suggested that the mechanical stress that acts on trees resulting from wind force tends to be maintained along the stems [17]. For branches that are not vertical, it has been shown that the mechanical stress from a branch's own weight remains relatively constant along the branch [18]. In the elastic similarity hypothesis, the deflection along a branch, occurring due to the load that acts on the branch, is assumed to be constant regardless of the length of the branch; *i.e.*, the deflection of the tip, *Δ*, divided by the length of the branch in question, *λ*, is a constant, regardless of how much *λ* may vary [8]. McMahon and Kronauer [8] derived the allometric relationship between the length (*x*) and diameter (*d*) of a branch from (i) the elastic similarity hypothesis (*i.e.*, *x*∝*d^α^*, where *α* = ⅔) and (ii) the uniform stress hypothesis (*i.e.*, *x*∝*d^α^*, where *α* = ½). They found that the actual allometric relationship was identical to the elastic similarity predictions (assuming a virtual tip in which the branch taper becomes zero distal to the real tip). Bertram [16] also looked at the allometric relationship between the length and the diameter of tree stems using the same models, but arrived at a different conclusion. He found that the distal stem elements of a tree can be disproportionately slender, such that the stem length, *x*, scales with respect to the stem diameter, *d*, in a manner that exceeds that predicted by the allometry of geometric self-similarity (*i.e.*, *x*∝*d^α^*, where *α* = 1), whereas the older elements of a tree trunk tend to scale in a manner that approximates elastic self-similarity predictions. Other studies suggest that the allometry of tree height and trunk taper progressively changes over the course of growth and development [19]–[21]. Niklas [19] suggested that trees comply with geometric self-similarity in their young portions and subsequently give the appearance of elastic or stress self-similarity as these portions age and become larger. However, which biomechanical model best reflects branches in nature is debatable.

The allometric relationships for these biomechanical hypotheses may be regarded as a rule for branch tapering. In the biomechanical models, the taper of a branch is expressed by one equation and is assumed to be smooth. However, real branches ramify, and it is reasonable to suppose that the diameter near the ramifying point deviates from the taper equation if biomechanical stress limits branch shape (because the weight that the branch must bear changes dynamically below and above the branching point). Assuming that one of the biomechanical hypotheses applies to tree branch architecture, the diameter needed to maintain the mechanical strength of a branch varies with branching angles and relative weights of distal branches. The relevant question is whether da Vinci's rule is strictly maintained for any realistic branching pattern when either of the biomechanical hypotheses is true. In other words, can da Vinci's rule be applied to biomechanically limited branches? Branches may not conform to da Vinci's rule when the diameter measurement points are restricted to positions that are near the branching point, which may be true for both biomechanical models.

In this study, we calculated the ratio of the cross-sectional areas of the upper and lower sides of a branching point of a branch using the two biomechanical models, and checked whether computed values matched da Vinci's rule. We also took field measurements of this ratio in real branches of *Fagus crenata* and *Abies homolepis* to examine whether da Vinci's rule is strictly maintained, and to check the predictions of the biomechanical models. On the basis of these data, we (i) discuss the question posed above, (ii) determine whether da Vinci's rule is biomechanically adequate, and (iii) determine whether da Vinci's rule is consistent with either of the two biomechanical models when applied to natural branching patterns.

## Methods and Materials

We used the daughter/mother ratio, *i.e.*, the ratio of the total cross-sectional area of the daughter branches to the cross-sectional area of the mother branch at the branching point, as an index for da Vinci's rule. Here, the daughter branches represent parts of a branch after ramification and the mother branch represents the part before ramification. If a tree obeys da Vinci's rule, the daughter/mother ratio must be 1.0 at any branching point of the tree.

### Calculation of the daughter/mother ratio using biomechanical models: Is da Vinci's rule consistent with biomechanical models?

#### A model for the uniform stress hypothesis

**Figure 1:**
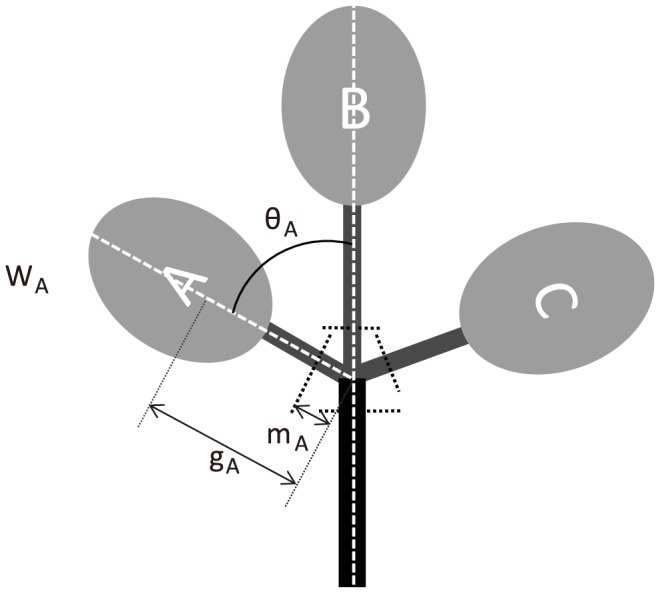
Overhead view of the proposed branching system scheme. A mother branch has several daughter branches, here labelled as A, B, and C. The branch's spatial arrangement is within a horizontal plane. The weight of daughter branches is defined as *W*_A_, *W*_B_, and *W*_C_ and the distances between the center of gravity and the base of each daughter are *g*_A_, *g*_B_, and *g*_C_. The branching angles of each daughter are *θ*_A_, *θ*_B_, and *θ*_C_ and the distances between the branching point and the measurement points are *m*_A_, *m*_B_, *m*_C_, and *m*_M_.

In our simulations, we considered a horizontal branch ramifying into *n* daughter branches within a horizontal plane, and calculations were performed for a virtual branch with an *n* of 2 or 3. Branches were assumed to be mostly influenced by their own weight. Each daughter was identified by *i* (*i* = A, B, etc.; Fig. 1). A branch's applied load was assumed to consist only of its own weight, which acts in the direction vertical to the plane in which the branch was arranged. We calculated the diameters of daughter and mother branches at the branching point based on the assumption that branches had a constant safety factor (uniform stress). We then calculated the daughter/mother ratio.

In the model, a branch was regarded as a horizontal beam loaded in the vertical direction. According to material mechanics, the relationship between the diameter and the load that acts on a beam at an arbitrary point can be represented as
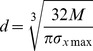
(1)
[22], where *M* is the moment determined by the magnitude of the load and the distance from the loading point, and *σ*_xmax_ is the maximum bending stress. Under the conditions of the uniform stress model, *σ*_xmax_ is constant, and the diameter depends only on the moment.

The moment that occurs at the branching point of the mother and daughter branches (*M*_M_ and *M_i_*, respectively) is
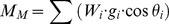
(2)

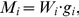
(3)where *W_i_* is the weight of daughter *i*, *g_i_* is the distance between the center of gravity of daughter *i* and the branching point, and *θ*_i_ is the branching angle of daughter *i*. When calculating the daughter/mother ratio, it is possible to set the measurement points of diameters as the branching point. However, when measuring in the field, there may be some distance between the branching point and the measurement point. Considering this, the moments can be rewritten as
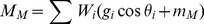
(2′)

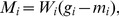
(3′)where *m*_M_ and *m_i_* are distances between the branching point and the measurement point of the mother or daughter *i*, respectively. In equation (2′), the section between the branching point and the measurement point on the mother branch is assumed to have little effect on *M*_M_. We ran the calculation applying zero and some probable values (1, 2, 3, and 5 cm) to *m*_M_ and *m*_i_. Substituting equation (2′) or (3′) into (1) gives the diameter of the mother and daughters. Applying these diameters, the cross-sectional area of the mother at the branching point can be obtained from
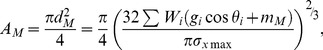
(4)where *d_M_* is the diameter of the mother branch. The total cross-sectional area of the daughters can be obtained from
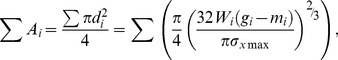
(5)where *d_i_* is the diameter of the daughter *i*. From equations (4) and (5), the daughter/mother ratio is
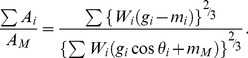
(6)According to equation (6), the daughter/mother ratio is independent of the maximum bending stress.

We calculated the daughter/mother ratio for various situations with different branching angles and daughter weights. The angles ranged from 0.1 to 90° and the daughter weights ranged from 1 to 10 kg. In simulations for branches where *n* equaled 3 (see the following results), the weight of the main daughter branch (as with branch B shown in Fig. 1) was set to 10 kg.

#### A model for the elastic similarity hypothesis

In the elastic similarity hypothesis, the deflection at the tip of a branch is assumed to be proportional to the overall length of the branch [8]. In other words, the deflection angle of an arbitrary microsection of a branch (with the constant relative length) is determined by its relative position on the branch, independent of branch length.

McMahon and Kronauer [8] assumed that the diameter in the vertical plane at a certain point is proportional to *s* ^3/2^ for a tapered beam, where *s* is the length of the section of the branch from the (selected) point to the tip. King and Loucks [14] also referred to the elastic similarity model and explained the relationship between diameter and length of a branch bending under its own weight as follows: for branches that maintain self-similarity, branch weight *W* is proportional to *d* ^2^ *l*, where *l* is the length of the branch, since *d* ^2^ *l* is proportional to branch volume. The moment arm is proportional to branch length. Therefore,
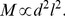
(7)If a branch is maintained in an elastically similar form, the relationship between curvature and length can be written as
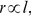
(8)where *r* is the radius of curvature of the branch at its base. According to the cantilever beam theory [22],
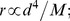
(9)substituting (9) and (7) into (8), the relationship between diameter and length is
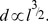
(10)In this study, we used the allometric relationships
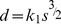
(a)

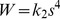
(b)

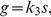
(c)which can be obtained from the above expressions, where *k*_1_, *k*_2_, and *k*_3_ are constants and *g* is the distance between the center of gravity and the branch base.

We now consider a straight horizontal branch without furcation, whose shape is similar to the form described by McMahon and Kronauer [8]. If determination of branch taper follows elastic similarity, the deflection angle of any section of the branch should be strictly maintained. According to cantilever beam theory, the deflection angle of an arbitrary microsection, d*θ*/d*s*, is expressed by the following equation:
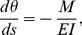
(11)where *M* is the moment due to the weight of the distal part of the branch, *E* is the modulus of elasticity or Young's modulus, and *I* is the second moment of area. For a circular cross-section, *I* is determined as
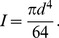
(12)For *η*(: = *s*/*λ*), where *λ* is whole length of the branch, the deflection angle of a microsection with a constant relative length is determined by
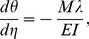
(13)and the moment that acts on the microsection due to the portion of the branch distal to the section is calculated as
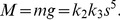
(14)Substituting (12), (14), and (a) into (13) gives
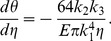
(15)
Equation (15) supplies information about the d*θ*/d*η* value that should be maintained in branches that keep elastic similarity. We propose that d*θ*/d*η* is maintained above and below the furcation, whether or not the taper equation is maintained locally around the branching point. If branching is taken into consideration, the moment that acts on the cross-section of the branch varies dramatically before and after furcation but varies smoothly in other positions that have no ramification. Therefore, the diameter should markedly change before and after branching if elastic similarity is maintained and must locally deviate from the taper equation.

Assuming that the distances between the point of diameter measurement and the branching point are represented by *m*_M_ and *m*_i_, we used equation (15) to determine d*θ*/d*η* for the microsection of the diameter measurement point on the mother branch. Subsequently, we considered a branch bearing several daughter branches (Fig. 1) and assumed that the longest daughter branch is a part of the main axis of the whole branch that includes the sections above and below the branching point; thus, the length of the longest daughter branch is a part of *λ*. The moment that occurs at the diameter measurement point on the mother branch (*M*_M_) can be obtained from equation (2′).

Substituting equations (12) and (15) into (13), setting (*s*_l_+*m*_M_) as *s* (: = *ηλ*), we obtain *d* ^4^ of the mother branch at the branching point required to maintain a constant d*θ*/d*η*:
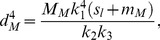
(16)where *s*_l_ is the length of the longest daughter branch. The square root of equation (16) is *d*_M_^2^, which can be used for calculating *A*_M_ ( = *πd*_M_^2^/4).

The sum of the cross-section areas of daughter branches is
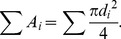
(17)From equations (17), (16), and (a), setting (*s*_i_−*m*_i_) as *s*, the daughter/mother ratio is expressed as
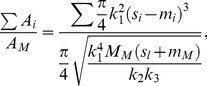
(18)where *s*_i_ is the length of each daughter branch. Substituting equations (2′), (b), and (c), equation (18) can be arranged as
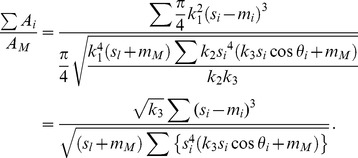
(19)Using equation (19), we calculated a daughter/mother ratio for a virtual horizontal branch with various branching forms. For *k*_3_, we used the value obtained from the allometric regression equation expressing the relationship between the data for branch length and the distance between the center of gravity of the branch and the branching point for *Fagus crenata* and *Abies homolepis*. These empirical data were obtained by methods described in the following section. If *k*_3_ is constant for any branch form, the daughter/mother ratio varies according to the length and branching angles of lateral daughters.

### Field measurements and evaluation of da Vinci's rule and each of the biomechanical models

Data on the diameters of several sections of branches, the lengths of the parts distal to these sections, the distances between the centers of gravity of the distal parts and the measurement section, and the moment due to each of the branch weights at each section were collected from lower branches of *Fagus crenata* (*ca.* 30 years old) and *Abies homolepis* (age unknown) specimens growing in the Nikko Botanical Garden (Nikko, Tochigi, Japan, E139°37′, N36°45′, mean annual temperature = 12.1°C, mean annual precipitation = 2400 mm); twigs were arranged in a horizontal plane. We subsequently explored the relationships between the length and the distance of the center of gravity of the distal part from the measurement section for each species, using exponential regressions. We used the least-squares method to fit the curves. The equations for the regression curves were used in the above model calculations.

We also collected data on the diameters of mother and daughter branches, the daughters' branching angles, and the number of daughters ramified from a mother branch, using other lower branches on the same trees. Thirteen branches from six *Fagus crenata* trees and 13 branches from seven *Abies homolepis* trees were measured. The heights of the sampled *Fagus crenata* trees and *Abies homolepis* trees ranged from 14.1 to 16.9 m (14.7±1.3 m, mean ± SD) and 9.4 to 22.6 m (13.8±5.5 m), respectively. The mean diameters at breast height were 26.4±1.5 cm and 31.6±13.1 cm, respectively. The average height at which the branches attached to the trunks was 1.5±0.4 m for *Fagus crenata* and 1.6±0.4 m for *Abies homolepis*. The length of the branches ranged from 1.8 to 6.3 m (3.2±1.4 m) for *Fagus crenata* and from 1.1 to 4.5 m (2.1±0.1 m) for *Abies homolepis*. The slope at the basal section of the *Fagus crenata* branches was 30.5±15.0°, which tended to become immediately gentle toward the tip along the branch, so that the slope of the measured section was 20.6±15.3°. For *Abies homolepis*, the slope at the basal section of the branches was 14.7±7.8°, with little variation along each branch. Several branching points (1–14 per branch) were measured on each branch. Diameters were measured both in the horizontal and vertical planes for each point. The maximum and minimum values of the diameter of mother branches were 56.0 and 8.3 mm for *Fagus crenata*, and 49.1 and 6.0 mm for *Abies homolepis*, respectively. The cross-sectional area for each point was then calculated as an ellipse and as a circle with a diameter obtained in the vertical plane. The ratio between the diameter in the vertical plane and the diameter in the horizontal plane was 1.065±0.007 for *Fagus crenata* and 1.034±0.003 for *Abies homolepis*. The average, maximum, and minimum values of the ratio between the cross-sectional area calculated as an ellipse and the area calculated as a circle with its diameter in the vertical plane were 0.943±0.006 (mean ± SE), 1.140, and 0.785 for *Fagus crenata*, and 0.970±0.003, 1.105, and 0.803 for *Abies homolepis*, respectively. We selected lower horizontal branches because this type of branch ramifies within a horizontal plane, as with the mechanical models above, simplifying the calculation for the biomechanical validity of branches. The daughter/mother ratio for each branching point was then calculated. The branches were located within a forest, and thus the effects of wind force were expected to be relatively small. The number of daughter branches ramified from a mother was always 2 in *Fagus crenata* and 2–5 in *Abies homolepis*. We measured 34 branching points for *Fagus crenata* and 39, 37, and 3 branching points in *Abies homolepis* with 2, 3, and more than 4 daughters, respectively. Branching points were weaker than other sections, and therefore thicker. We chose a measurement point located where the thicker section ended. As a result, the measurement points were 1–8 cm away from the branching point, despite our efforts to minimize the distance between branching and measurement points. The larger the mother branch, the larger the required distance was between the measurement point and the branching point. The effect of this distance was assumed to be negligible because the distance was small compared with the overall size of the branches.

The above biomechanical model calculations refer to the difference between the weight of the main branch and the lateral daughters. Therefore, an index that defines the weight difference between the daughters is required for analysis of the measured data. We defined this difference as the daughters' degree of deviation and described it as
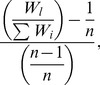
(20)where *W*_l_ is the weight of the largest daughter branch and *n* is the number of daughters ramified from one mother branch. The numerator indicates the difference between the ratio of the weight of the largest daughter branch to the total weight of the daughter branches (the value of which is restricted into 1/*n*; 1 because it is the value related to the largest daughter branch) and the ratio for the situation in which all daughter branches have the same weight, which, in effect, represents the extent of deviation from the uniformity of the weight of daughter branches. This value can vary from zero to (*n*−1)/*n*. It was standardized by dividing by (*n*−1)/*n* so that the upper limit of the value of the daughters' degree of deviation becomes 1.0. Therefore, the daughters' degree of deviation has the following characteristics. When one of the daughter branches is much larger than the others, the degree approaches 1. When a branch does not ramify, *i.e.*, the weights of the other branches are zero, the value is 1. In contrast, when there is no difference between the weights of daughter branches, the degree is zero. The weight of each daughter branch was determined from the regression equation already calculated from field data.

The validity of the uniform stress model was also examined. If a branch obeys the uniform stress model, the relationship between daughter and mother branches can be determined as follows. According to the uniform stress model, the relationship between the moment and diameter at any point on a branch is described by equation (1). Combining equations (2), (3), and (1), the relationship between the diameter of the mother branch and the diameters of daughter branches is
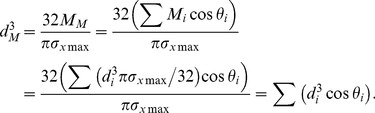
(21)From this, ∑ (*d_i_* ^3^cos*θ_i_*)/*d*_M_^3^ = 1 is expected if the maximum bending stress is constant along the branch. We used the left side of this equation as an index for the uniform stress model and examined the validity of the elastic similarity model using the following equation, derived from equations (2′), (11), and (12):
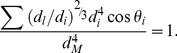
(22)The left side of equation (22) was used as the index for the elastic similarity model. For simplicity, the distance between the branching point and the measurement point was not considered in these calculations.

We performed statistical analyses to assess the relationship between the cross-sectional area of the mother branch and the sum of the cross-sectional areas of the daughter branches, the relationship between the daughters' degree of deviation and the daughter/mother ratio, and the relationship between the daughters' degree of deviation and the index for each of the biomechanical models. Correlation coefficients were calculated for these relationships with MS Excel 2010. Linear regressions were also performed using the least-squares method for each relationship.

Using the data on diameter and branching angle of each daughter branch, we also estimated daughter/mother ratios, assuming that the branches follow one of the two mechanical models. If a branch were to follow the uniform stress model, the daughter/mother ratio would be calculated using equation (6). Assuming that a branch obeys the elastic similarity model, the daughter/mother ratio can also be calculated using the equation derived from equations (19) and (a). For simplicity, we describe the equation for which *m*_i_ and *m*_M_ are zero, resulting in
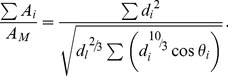
(23)Subsequently, we compared each of the estimated daughter/mother ratios with the actual daughter/mother ratio using the method proposed by Kozak and Smith [23]. A measure of mean bias, *B* (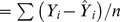), and the standard error of estimation, SEE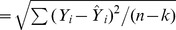, were calculated for each model, where *Y*_i_ is the actual observation of the dependent variable, 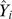 is the predicted value of the actual observation, n is the number of observations, *k* is the number of estimated parameters used in the estimation, and 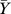 is the average of the actual observations. For completeness and to determine the magnitude of the effects of *m*_i_ and *m*_M_, we also calculated the daughter/mother ratio for each biomechanical model with equations (6) or (19), using the actual *m*_i_ and *m*_M_ values, and applied the allometric relationships between diameter and length, between diameter and the distance from the base to the center of gravity, and between diameters and weights of branches and *k*_3_ (all obtained from the branch group that excluded those branches used for determining daughter/mother ratios). We also determined the daughter/mother ratio excluding the bark in an indirect manner, using the relationship between diameter and bark thickness obtained from the branch group that excluded branches used for determining daughter/mother ratios. The daughter/mother ratio obtained in this indirect manner is less reliable than other measures because it includes several indirect elements. Nevertheless, the rough estimates proved useful in our considerations of magnitudes of effects.

### Strain gauge measurements for exploring the uniform stress hypothesis

We measured the strains on branches caused by their own weights. Strains were measured in three branches of *Fagus crenata* and four branches of *Abies homolepis* at several points along the longitudinal axis of each branch. The branches used for this measurement were all first order branches and were selected from the branches used for the above measurement. Branches 1, 2, and 3 of *Fagus crenata* had lengths of 6, 2.5, and 6.3 m, respectively, and branches 1, 2, 3, and 4 of *Abies homolepis* had lengths of 4.8, 4.5, 1.8, and 5 m, respectively. The mean, maximum, and minimum values of the diameters of the measurement points were 3.6±1.4 (mean ± SD), 6.1, and 1.2 cm in *Fagus crenata*, and 3.4±1.5, 5.8, and 1.2 cm in *Abies homolepis*, respectively.

Strain gauges (FLA-5-11, Tokyo Sokki Kenkyujo, Tokyo, Japan) were used to detect changes in strain. We used cyanoacrylate adhesive (CN, Tokyo Sokki Kenkyujo, Tokyo, Japan) to attach the strain gauges to the decorticated surfaces of the upper and lower sides of each point of the branches prior to their removal from the trunks. The strain gauges were connected to a multi-recorder (TMR-200, Tokyo Sokki Kenkyujo, Tokyo, Japan) in such a way as to allow the upward deflection of a branch to be converted to a negative strain value. Once the branches were fixed to the trunks, the strain values were set to zero. The branches were then cut down at the fixed end and were laid sideways to be freed from their own weight. Infinitesimal changes in the strain values were detected and recorded by the multi-recorder during this treatment. For these measurements, we used the half-bridge method, which uses a bridge circuit made up of two strain gauges and outputs the difference between the values of the strain at the upper and lower sides of each measurement point. One-half of the original value was treated as an estimate of the strain value for the upper surface. This method is suitable for measuring the bending stress because the tensile component is eliminated. The strain at the surface of the branch is proportional to the stress at that point if Young's modulus of the sapwood is constant. Therefore, the uniform stress model can be assessed from the strain data. The variation in strain caused by a branch's own weight along each branch was investigated.

No permits were required for this study, which complied with all relevant regulations.

## Results

### Differences in daughter/mother ratios between each of the biomechanical models and da Vinci's rule, estimated by model calculations

**Figure 2:**
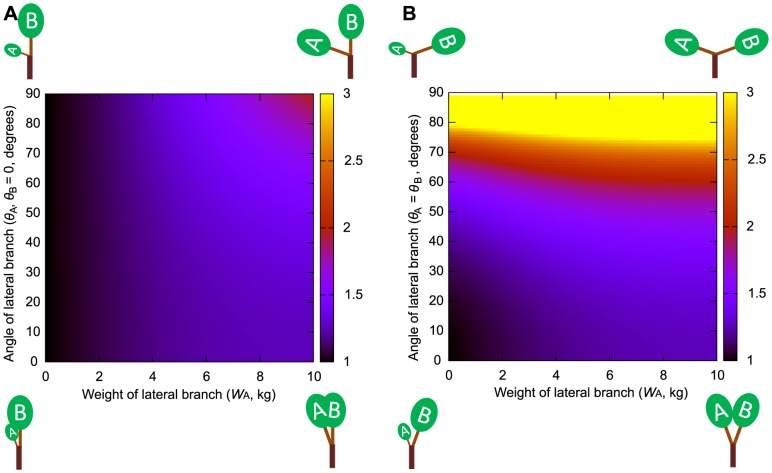
Daughter/mother ratios based on assumptions of the uniform stress model for two daughters. Daughter/mother ratios based on assumptions of the uniform stress model plotted in palette maps. The range of values for daughter/mother ratios is represented by a range of colors. Values greater than 3 are shown in yellow. This plot shows two daughters (A and B) with weights denoted by *W*_A_ and *W*_B_. The weight of daughter B is fixed at 10 kg, and the weight of daughter A is set to vary from 0 to 10 kg. *m*_M_ and *m_i_* are 1 cm. (A) *θ*_B_ was fixed at zero, *θ*_A_ was set to vary from 0.1 to 90°. (B) The angles of daughter A and B are set as *θ*_A_ = *θ*_B_ and to vary from 0.1 to 90°.

**Figure 3:**
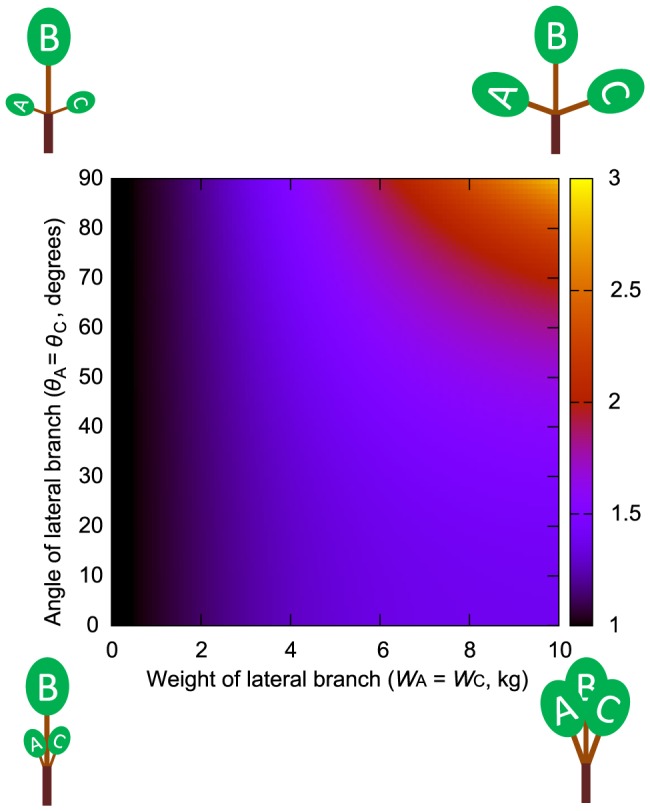
Daughter/mother ratios based on assumptions of the uniform stress model for three daughters. Daughter/mother ratios based on assumptions of the uniform stress model for three daughters: A, B, and C. Different values of the daughter/mother ratio are represented by different colors. Values greater than 3 are shown in yellow. The angles of daughters A and C are set as *θ*_A_ = *θ*_C_ and to vary from 0.1 to 90°. The weight of daughter B is fixed at 10 kg, and the weights of daughters A and C are set as *W*_A_ = *W*_C_ and to vary from 0 to 10 kg. *m*_M_ and *m_i_* are 1 cm.

After applying various values to the branching angles and weights of daughter branches, using the uniform stress model, we found that the daughter/mother ratio was always >1.0 if *m*_M_ and *m_i_* were assumed to be zero. However, the daughter/mother ratio generally decreased when *m*_M_ and *m_i_* were set to larger values. The daughter/mother ratio increased when the weights of the lateral daughter branches (which grow in different directions relative to the mother branch) relative to the weight of the main daughter branch (which has a branching angle of zero) increased (Figs. 2, 3 and Tables S1, S2, S3). This tendency was amplified when lateral daughter branching angles were increased (Figs. 2, 3). This occurred because the daughter/mother ratio also increased with the branching angles of lateral daughter branches, as the moment of the mother branch was influenced by the cosine of each daughter's branching angle.

For any ratio of the total lateral daughters' weights and the main daughter's weight, the minimum value of the daughter/mother ratio can be found when the branching angles of the daughters are near zero (Figs. 2, 3). The larger the weight of the main daughter relative to that of the lateral daughters is, the lower the minimum value of the daughter/mother ratio is. For example, when the number of daughter branches was 2 (and one of them was the main daughter) and *m*_M_ and *m_i_* were assumed to be 1.0, the minimum value of the ratio was 1.24 for *W*_A_∶*W*_B_ = 1∶1. When the ratio between the main and the lateral daughters was 10∶1, the minimum daughter/mother ratio fell to 1.04, which can be treated as roughly equal to 1 (Fig. 2A and Table S1). When assuming a branch with two daughters and neither of them was a main daughter, the ratio was larger than the above values (Fig. 2B and Table S2). When the weights of the branches were fixed, the daughter/mother ratio rose with increasing daughter branching angles, gently in the range of 0–60°, and swiftly in the range of 80–90° (Fig. 2B).

When the number of daughters was three or more, the value of the daughter/mother ratio was generally larger than the above values calculated for the branching points with one main daughter and one lateral daughter (Fig. 3 and Table S3). In Fig. 3, the daughters consisted of one main daughter and several lateral daughters. However, it is also possible to have no main daughter. In such a situation, the daughter/mother ratio should be larger because the rate of the moment that the mother branch must bear, originating from lateral daughter branches (reduced by the cosine effect), is larger than when a branch has a main daughter.

It should be noted that values may be overestimated when there is poor balance with respect to the mother axis between daughter branches A and B or between daughter branches A and C (e.g., the upper left corner of Fig. 2B) because the actual branch must bear the shear stress due to torsion. If the daughters are balanced or the main daughter is sufficiently heavier than other daughters, the shear stress is likely to be negligible. In reality, branches that have extremely unbalanced daughter branches were rarely observed and therefore, this would not be a serious problem for branches in nature.

**Figure 4:**
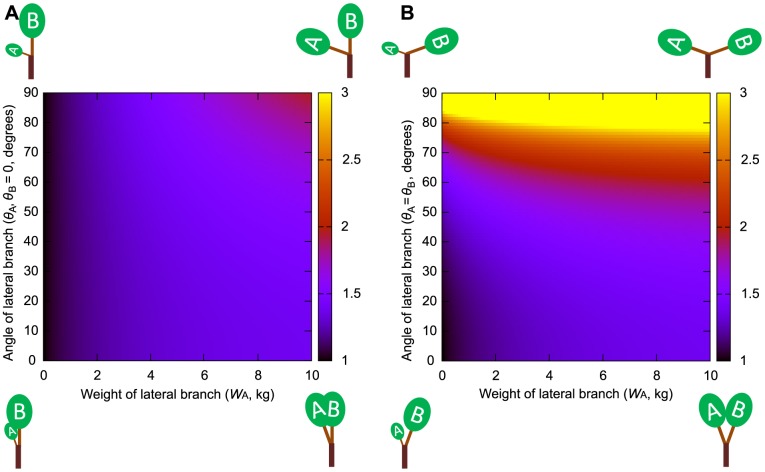
Daughter/mother ratios based on assumptions of the elastic similarity model for two daughters. Daughter/mother ratios based on the assumptions of the elastic similarity model for two daughters, A and B, with weights *W*_A_ and *W*_B_. Different values of the daughter/mother ratio are represented by different colors. Values greater than 3 are shown in yellow. The weight of daughter B is fixed at 10 kg and the weight of daughter A is set to vary from 0 to 10 kg. (A) *θ*_B_ was fixed at zero, *θ*_A_ was set to vary from 0.1 to 90°. (B) The angles of daughter A and B are set as *θ*_A_ = *θ*_B_ and to vary from 0.1 to 90°.

**Figure 5:**
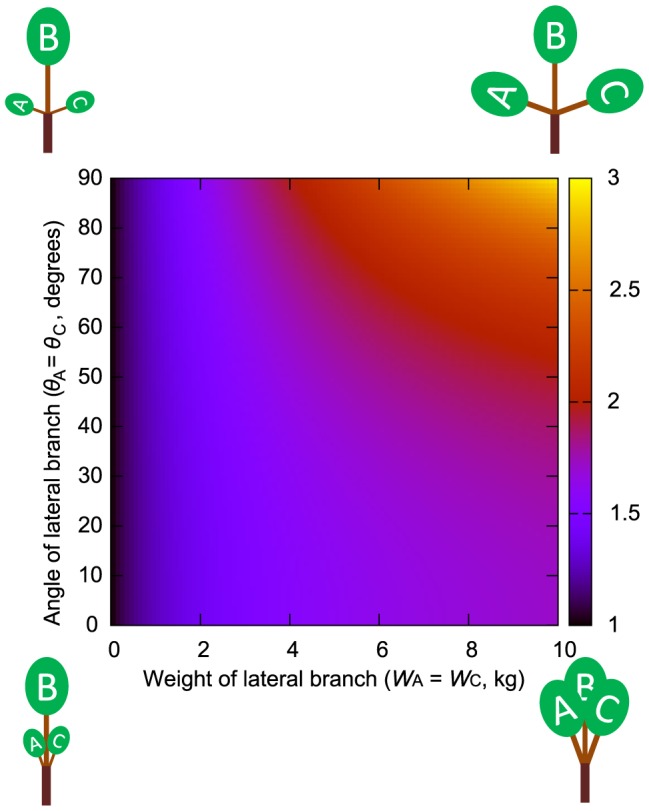
Daughter/mother ratios based on assumptions of the elastic similarity model for three daughters. Daughter/mother ratios based on assumptions of the elastic similarity model for three daughters, A, B, and C. Different values of the daughter/mother ratio are represented by different colors. Values greater than 3 are shown in yellow. The angles of daughters A and C are set as *θ*_A_ = *θ*_C_ and to vary from 0.1 to 90°. The weight of daughter B is fixed at 10 kg, and the weights of daughters A and C are set as *W*_A_ = *W*_C_ and to vary from 0 to 10 kg. *m*_M_ and *m_i_* are 1 cm.

The constants needed for elastic similarity model calculations obtained from field measurements were *k*_2_ = 4.91×10^−11^ and *k*_3_ = 0.3932 for *Fagus crenata* and *k*_2_ = 3.93×10^−10^ and *k*_3_ = 0.4397 for *Abies homolepis*. The change in the value of the daughter/mother ratio obtained from the elastic similarity model calculations using these values was similar to that obtained from the uniform stress model (Figs. 4, 5 and Tables S4, S5, S6). However, the absolute value of the daughter/mother ratio obtained from the elastic similarity model was larger under the same conditions for a branch with three daughters. The daughter/mother ratio generally decreased when *m*_M_ and *m_i_* were set to large values, whereas the trend mentioned above was maintained when *m*_M_ and *m*_i_ were set to large values or changed by the same method as that used for the measurement point choice we established in our field observations. Estimated values for the elastic similarity model might also deviate from the actual value due to shear stress when there is poor balance with respect to the mother axis between the daughter branches A and B or daughter branches A and C.

### Comparison between actual and theoretical values of the daughter/mother ratio; validation of the biomechanical models in *Fagus crenata* and *Abies homolepis*

**Figure 6:**
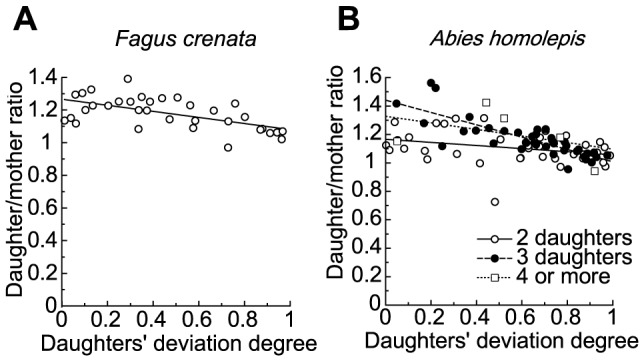
Daughter/mother ratios vs. daughters' degrees of deviation. Daughter/mother ratios vs. daughters' degree of deviation in (A) *Fagus crenata* and (B) *Abies homolepis*. The number of daughters branching from a mother branch is two in *Fagus crenata*, and two (open circles), three (filled circles), and four to five (open squares) in *Abies homolepis*.

**Figure 7:**
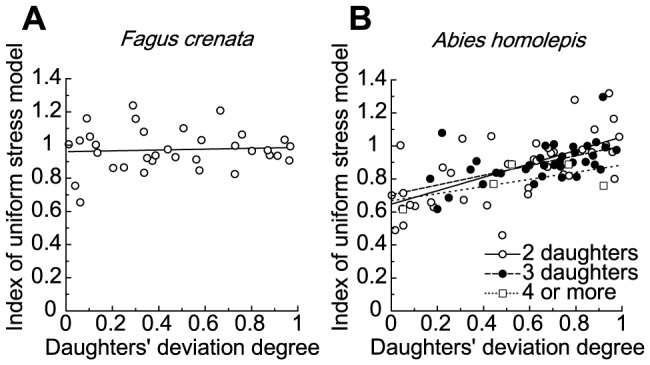
Indices of the uniform stress model vs. daughters' degrees of deviation. Indices of the uniform stress model vs. daughters' degrees of deviation in (A) *Fagus crenata* and (B) *Abies homolepis*. The theoretical value of the index is 1.0, independent of daughters' degrees of deviation.

The sum of the cross-sectional areas of daughter branches was a little larger than the cross-sectional area of the mother branch at most branching points in *Fagus crenata* and at some branching points in *Abies homolepis*, whereas the daughter/mother ratios were not so much away from 1.0 (Fig. 6). The daughter/mother ratio for these branching points became larger as the daughters' degree of deviation became smaller, but this trend was very weak for branching points with two daughters in *Abies homolepis*, and the value was very close to the value obtained when using da Vinci's rule for branching points with two daughters in *Abies homolepis* (Fig. 6, Table 1). This tendency for negative correlation did not change whether the cross-sectional area was calculated as an ellipse or a circle, and appeared to roughly coincide with the two biomechanical model predictions rather than with da Vinci's rule. Indeed, ∑ (*d_i_* ^3^cos*θ_i_*)/*d*_M_^3^, the index used for the uniform stress model, was almost 1.0 (0.97±0.02 SE), regardless of the daughters' degree of deviation in *Fagus crenata* (Fig. 7A, *r* = 0.06). Thus, the stress uniformity seems to explain the branch form near branching points in *Fagus crenata*. However, in *Abies homolepis*, the value of the index deviated somewhat from the theoretical value (0.88±0.02; Fig. 7B). Here, contrary to our expectations, ∑ (*d_i_* ^3^cos*θ_i_*)/*d*_M_^3^ increased with increasing daughters' degree of deviation in *Abies homolepis* (Fig. 7B, Table 1). Therefore, the uniform stress model does not apply well to *Abies homolepis* branches. A difference in the trend of the daughter/mother ratio among the groups of branching points with different number of daughter branches (*n*) was observed. In *Abies homolepis*, the slope of the relationship between the daughter/mother ratio and the daughters' degree of deviation increased with *n*. On the other hand, the slope of ∑ (*d_i_* ^3^cos*θ_i_*)/*d*_M_^3^ decreased with *n*.

**Table 1:** Constants of the regression equations (*y* = A*x*+B) and correlation coefficients (*r*) in Figs. 6, 7, and 8.

	Daughter/mother ratio			Index of uniform stress model			Index of elastic similarity model		
	A	B	r	A	B	r	A	B	r
***Fagus crenata***									
**two daughters**	−0.19	1.27	−0.59	0.03	0.96	0.06	0.19	0.79	0.39
***Abies homolepis***									
**two daughters**	−0.11	1.16	−0.32	0.41	0.65	0.65	0.64	0.48	0.69
**three daughters**	−0.43	1.44	−0.81	0.30	0.70	0.58	0.48	0.53	0.67
**four or more**	−0.23	1.33	−0.42	0.21	0.67	0.64	0.41	0.45	0.78

*x* represents the daughters' degree of deviation, and *y* represents the daughter/mother ratio or the index of each biomechanical model.

**Figure 8:**
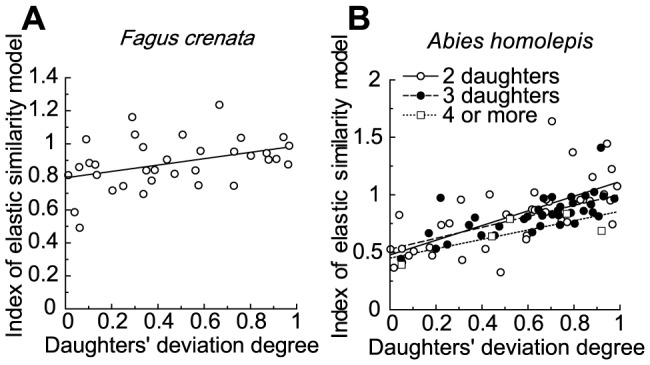
Elastic similarity model indices relative to daughters' degrees of deviation. Elastic similarity model indices plotted against daughters' degrees of deviation in (A) *Fagus crenata* and (B) *Abies homolepis*. The theoretical value of the index is 1.0 independent of daughters' degrees of deviation.

The index for the elastic similarity model became smaller than 1.0 as the daughters' degree of deviation neared zero and generally took on a value smaller than the index for the uniform stress model in both species (Fig. 8, Table 1), therefore deviating more from the theoretical value than the uniform stress model. In *Fagus crenata*, there appeared to be little correlation between the index for the elastic similarity model and the daughters' degree of deviation (average: 0.88±0.03, mean ± SE). However, in *Abies homolepis*, there was a positive correlation between these variables, particularly in branches with two daughters; this was also the case for the uniform stress model index (Table 1). These results indicate that the uniform stress model represented real branches in *Fagus crenata*, but neither the mechanical models nor da Vinci's rule represented *Abies homolepis* branches.

Statistical analyses of predicted and measured daughter/mother ratios showed that the measured ratio was smaller than the predicted ratio in both mechanical models and was larger than the value obtained with da Vinci's rule ( = 1; Table 2). Analyses also showed that SEE was smallest for the uniform stress model in *Fagus crenata* and for branching points with three or more daughters in *Abies homolepis* (the measured daughter/mother ratio value was nearest to the prediction of the uniform stress model in *Fagus crenata* and for branching points with three or more daughters in *Abies homolepis*). However, for branching points with two daughters in *Abies homolepis*, SEE was smallest for the value obtained with da Vinci's rule. Thus, we suggest that branches in *Fagus crenata* and branching points with three or more daughters in *Abies homolepis* comply with the uniform stress model, whereas branching points with two daughters in *Abies homolepis* comply with da Vinci's rule. When excluding bark thickness, the daughter/mother ratio for *Fagus crenata* became slightly larger, but our conclusions remained almost unaffected. As an exception, the exclusion of bark thickness in *Abies homolepis* from the daughter/mother ratio decreased the value (it approached 1.0 in both branching points with two daughters and branching points with three or more daughters) and decreased SEE for da Vinci's rule (Table 2). Therefore, it is possible that branching points with three daughters in *Abies homolepis* best comply with da Vinci's rule. For branching points with four or more daughters in *Abies homolepis*, SEE (excluding bark thickness) was smallest in the elastic similarity model, indicating the possibility that these branching points best comply with the elastic similarity model. The daughter/mother ratios calculated by the biomechanical models from the measured diameters of daughter branches when including *m*_i_ and *m*_M_ were slightly smaller than the ratios calculated without them (in both species), but the difference was very small and did not change our conclusions.

**Table 2:** Biases and standard errors of estimates (SEE) of daughter/mother ratios estimated in compliance with the assumptions of each model.

	da Vinci's rule		Uniform stress model		Elastic similarity model	
	Bias	SEE	Bias	SEE	Bias	SEE
***F. crenata***						
**two daughters**	0.180 (0.143)	0.206 (0.178)	−0.072 (−0.062)	0.120 (0.123)	−0.134 (−0.090)	0.183 (0.165)
***A. homolepis***						
**two daughters**	0.114 (0.040)	0.159 (0.113)	−0.118 (−0.156)	0.295 (0.252)	−0.176 (−0.109)	0.279 (0.237)
**three daughters**	0.168 (0.028)	0.216 (0.102)	−0.108 (−0.212)	0.160 (0.274)	−0.160 (−0.106)	0.232 (0.197)
**four or more**	0.216 (0.059)	0.324 (0.149)	−0.130 (−0.227)	0.168 (0.286)	−0.170 (−0.084)	0.224 (0.126)
**Total**	0.141 (0.037)	0.190 (0.110)	−0.119 (−0.182)	0.251 (0.259)	−0.177 (−0.113)	0.271 (0.229)

Values in parentheses are estimates obtained from calculations that take *m*_i_ and *m*_M_ into consideration for the uniform stress model and elastic similarity model, and take bark thickness into consideration for da Vinci's rule.

### Additional validation of the uniform stress model using measurements of strain caused by the weights of branches

**Figure 9:**
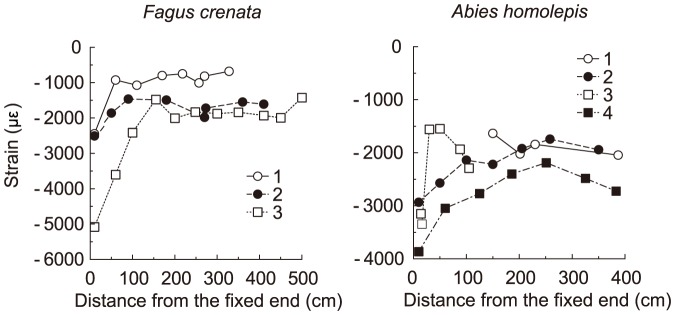
Changes in strain values of branches after bending, freed from their own weights. Changes in strain values (με) for three branches of *Fagus crenata* and four branches of *Abies homolepis* after being freed from their own weights, plotted against the distance from the fixed end of each branch. The lines are drawn to connect the points of each branch. Legends show individual numbers of each branch.

The changes in the strain value (με) tended to be smallest near the fixed end, which indicates that the degree of deflection caused by a branch's own weight is largest at the fixed end (Fig. 9). For *Fagus crenata*, the strain value increased with distance from the fixed end and settled in a constant range (correlation coefficients excluding the data for the position close to the fixed end were *r* = 0.20, 0.06, and 0.02 for *Fagus crenata* branches 1, 2, and 3, respectively). Likewise, for *Abies homolepis*, the value increased with increasing distance from the fixed end and became constant after the distance from the fixed end exceeded approximately 1 m. The strain values ranged from approximately −1000 to approximately −2000 με in *Fagus crenata* and −1500 to −2500 με in *Abies homolepis*, excluding the segments close to the fixed end. At the segment closest to the fixed end, the values were −5,000 or −2500 με in *Fagus crenata* and −3000, −3500, and −4000 με in *Abies homolepis*.

## Discussion

From the biomechanical model calculations, we demonstrated that the daughter/mother ratio is influenced by differences among daughter weights and branching angles and may deviate from 1.0 when the weights or branching angles of lateral daughter branches are relatively large (when mechanical limitations dominate tree design). For a common branching occurrence in nature where the main daughter is much larger than lateral daughters, the ratio may be close to 1.0. In such a case, the maintenance of mechanical stability or safety may keep the value near 1.0, resulting in agreement with da Vinci's rule. The daughter/mother ratio value can also be close to 1.0 when the lateral daughters' branching angles are small. In practice, the angles are 50–80° for *Abies homolepis* and 10–50° for *Fagus crenata*. These values are sufficient (i) to produce a bending point such that the daughter/mother ratio is far more than 1.0 and (ii) for determining whether branching points best fit da Vinci's rule or one of the biomechanical models.

In *Fagus crenata* field measurements, diameters measured at points before and after branching were in agreement with the uniform stress model. The elastic similarity model was less congruent with empirical measurements, but the indices of both models were close to 1.0. This indicates that horizontal branches of *Fagus crenata* fit the uniform stress model best, the elastic similarity model second, and da Vinci's rule least well. The strains resulting from the branches' own weights were almost constant in *Fagus crenata*, excluding the segments close to the fixed end (Fig. 9). This provides good support for the uniform stress model (branching was rarely observed close to the fixed end). However, for *Abies homolepis*, the daughter/mother ratio closely followed da Vinci's rule for branching points with two daughters. In this species, indices of the two models changed with the daughters' degree of deviation, contrary to our expectations. Furthermore, the daughter/mother ratio estimated without bark followed da Vinci's rule better for branching points with two or three daughters, although the increasing trend with decreasing daughters' degree of deviation was minimal for branching points with three daughters. Thus, it seems that the architecture of *Abies homolepis* branches near branching points is forced to maintain a positive da Vinci's rule index rather than to conform to biomechanical safety or stability. However, the strain variations along branches caused by the branches' own weights were minimal in *Abies homolepis*, other than in segments close to the proximal end, indicating that branches of this species maintain uniformity of mechanical safety (Fig. 9).

A possible explanation for this contradiction in *Abies homolepis* may be the heterogeneity of wood properties. The relationship between maximum bending stress and diameter at a point on a branch may vary with the mechanical properties of wood. Reaction wood (tension wood and compression wood) is produced in inclined trunks, and differs from normal wood in mechanical properties [24]. The theoretical values we calculated assumed that the wood was uniform in its properties. It is possible that the reaction wood content in branches influenced their mechanical properties and diameters; under these circumstances, there may be simultaneous compliance with da Vinci's rule and a uniformity of stress. Additionally, branch shedding may also have affected results. In young *Abies homolepis* branches, the number of daughters ramifying from one mother branch was usually three and sometimes four or more, and branching points with two daughters may have experienced cladoptosis. Sone et al. [6] showed that only branches that have experienced shedding coincide with da Vinci's rule in *Acer rufinerve* and suggested that shedding is necessary to maintain da Vinci's rule in branching architecture. Such a branch may have established its architecture according to mechanical constraints before shedding, at which time it did not coincide with da Vinci's rule, and then shed several daughter branches of a certain cross-sectional area so that the branch diameters temporarily would coincide with da Vinci's rule. The diameters of such branches may be modified gradually if mechanical constraints exist, and there may be many branches in various states of modification in nature. Thus, it seems natural that a scattering of index values will occur after cladoptosis. However, without bark, the estimated daughter/mother ratio fit da Vinci's rule well, for most branching points. Therefore, it is reasonable to suppose that a branch of *Abies homolepis* will comply with da Vinci's rule regardless of the number of daughter branches.

The uniform stress model predicted the daughter/mother ratio better than the elastic similarity model in both species. Therefore, we propose that the branches we measured were more consistent with the uniform stress hypothesis than with the elastic similarity hypothesis.

Da Vinci's rule does not define the locations at which cross-sectional areas should be measured. The daughter/mother ratio measured at points nearest the branching point often deviated from 1.0, but the deviation was moderate and the ratio could be 1.0 if measurements were made farther from the branching point. The daughter/mother ratios calculated by the biomechanical models followed the same trend, and similar ratio values were predicted. Thus, Leonardo da Vinci's rule does not rule out compliance with biomechanical models in realistic circumstances. Similarly, the models do not preclude compliance with da Vinci's rule, although predictions of the two models and da Vinci's rule are not identical.

The method used here can also be applied to branches that are not horizontal, and can be modified to include the mechanical stress caused by wind, provided that the displacement of the branch is negligible. Indeed, the mechanical stress due to dynamic loads such as wind and snow is an important factor in a tree's mechanical safety [17], [25]–[26]. The biomechanical calculations can be applied to any branch form by decomposing the load acting on the branch, whatever the cause, into three-dimensional elements (two vertical and one horizontal). Further verification of various branch shapes requires additional data on the mechanical state of branches in nature.

We did not investigate tree branching in terms of hydraulic ability which is also a possible factor limiting tree morphology in this study. Further studies are required to clarify the relationship between hydraulic ability and tree branching.

## Supporting Information

Table S1 **Numerical data of** **Fig. 2A** **.**
(DOC)

Table S2 **Numerical data of** **Fig. 2B** **.**
(DOC)

Table S3 **Numerical data of** **Fig. 3** **.**
(DOC)

Table S4 **Numerical data of** **Fig. 4A** **.**
(DOC)

Table S5 **Numerical data of** **Fig. 4B** **.**
(DOC)

Table S6 **Numerical data of** **Fig. 5** **.**
(DOC)
