# Supplementary material for: Tree Branching: Leonardo da Vinci's Rule versus Biomechanical Models
Source: PLoS One. 2014 Apr 8;9(4):e93535. doi: 10.1371/journal.pone.0093535 (PMC3979699; doi:10.1371/journal.pone.0093535)
Supplement: Table S1 — Numerical data of Fig. 2A . (DOC) [file pone.0093535.s001.doc]

Table S1. Numerical data of Fig. 2A.

|  | **Weight of daughter A (kg)** | | | | | | | | | | |
| --- | --- | --- | --- | --- | --- | --- | --- | --- | --- | --- | --- |
| ***θ*_A_ (degrees, *θ*_B_ = 0)** | **0** | **1** | **2** | **3** | **4** | **5** | **6** | **7** | **8** | **9** | **10** |
| **0** | 1.00 | 1.04 | 1.09 | 1.13 | 1.17 | 1.20 | 1.22 | 1.24 | 1.25 | 1.26 | 1.26 |
| **10** | 1.00 | 1.04 | 1.09 | 1.13 | 1.17 | 1.20 | 1.23 | 1.25 | 1.26 | 1.26 | 1.26 |
| **20** | 1.00 | 1.04 | 1.09 | 1.14 | 1.18 | 1.21 | 1.24 | 1.26 | 1.27 | 1.28 | 1.28 |
| **30** | 1.00 | 1.04 | 1.09 | 1.14 | 1.18 | 1.22 | 1.25 | 1.28 | 1.30 | 1.31 | 1.32 |
| **40** | 1.00 | 1.04 | 1.09 | 1.15 | 1.20 | 1.24 | 1.28 | 1.31 | 1.33 | 1.35 | 1.37 |
| **50** | 1.00 | 1.04 | 1.10 | 1.15 | 1.21 | 1.26 | 1.31 | 1.35 | 1.38 | 1.41 | 1.43 |
| **60** | 1.00 | 1.04 | 1.10 | 1.16 | 1.23 | 1.29 | 1.35 | 1.40 | 1.45 | 1.49 | 1.52 |
| **70** | 1.00 | 1.04 | 1.10 | 1.17 | 1.25 | 1.32 | 1.39 | 1.46 | 1.52 | 1.58 | 1.64 |
| **80** | 1.00 | 1.04 | 1.11 | 1.19 | 1.27 | 1.35 | 1.44 | 1.53 | 1.62 | 1.71 | 1.79 |
| **90** | 1.00 | 1.04 | 1.11 | 1.20 | 1.29 | 1.39 | 1.50 | 1.62 | 1.74 | 1.86 | 2.00 |
