# Supplementary material for: Tree Branching: Leonardo da Vinci's Rule versus Biomechanical Models
Source: PLoS One. 2014 Apr 8;9(4):e93535. doi: 10.1371/journal.pone.0093535 (PMC3979699; doi:10.1371/journal.pone.0093535)
Supplement: Table S2 — Numerical data of Fig. 2B . (DOC) [file pone.0093535.s002.doc]

Table S2. Numerical data of Fig. 2B.

|  | **Weight of daughter A (kg)** | | | | | | | | | | |
| --- | --- | --- | --- | --- | --- | --- | --- | --- | --- | --- | --- |
| ***θ*_A_ (degrees, =*θ*_B_)** | **0** | **1** | **2** | **3** | **4** | **5** | **6** | **7** | **8** | **9** | **10** |
| **0** | 1.00 | 1.04 | 1.09 | 1.13 | 1.17 | 1.20 | 1.22 | 1.24 | 1.25 | 1.26 | 1.26 |
| **10** | 1.01 | 1.05 | 1.10 | 1.14 | 1.18 | 1.21 | 1.24 | 1.25 | 1.26 | 1.27 | 1.27 |
| **20** | 1.04 | 1.08 | 1.13 | 1.18 | 1.22 | 1.25 | 1.28 | 1.29 | 1.30 | 1.31 | 1.31 |
| **30** | 1.10 | 1.14 | 1.19 | 1.25 | 1.29 | 1.32 | 1.35 | 1.37 | 1.38 | 1.38 | 1.38 |
| **40** | 1.19 | 1.24 | 1.30 | 1.35 | 1.40 | 1.43 | 1.46 | 1.48 | 1.49 | 1.50 | 1.50 |
| **50** | 1.34 | 1.39 | 1.46 | 1.52 | 1.57 | 1.61 | 1.64 | 1.67 | 1.68 | 1.69 | 1.69 |
| **60** | 1.58 | 1.65 | 1.72 | 1.79 | 1.86 | 1.91 | 1.94 | 1.97 | 1.98 | 1.99 | 2.00 |
| **70** | 2.04 | 2.12 | 2.22 | 2.31 | 2.39 | 2.45 | 2.50 | 2.53 | 2.55 | 2.57 | 2.57 |
| **80** | 3.20 | 3.32 | 3.47 | 3.62 | 3.74 | 3.84 | 3.92 | 3.97 | 4.00 | 4.02 | 4.03 |
| **90** | 95.08 | 93.28 | 93.95 | 95.77 | 98.29 | 101.29 | 104.62 | 108.19 | 111.94 | 115.82 | 119.79 |
