# Supplementary material for: Tree Branching: Leonardo da Vinci's Rule versus Biomechanical Models
Source: PLoS One. 2014 Apr 8;9(4):e93535. doi: 10.1371/journal.pone.0093535 (PMC3979699; doi:10.1371/journal.pone.0093535)
Supplement: Table S3 — Numerical data of Fig. 3 . (DOC) [file pone.0093535.s003.doc]

Table S3. Numerical data of Fig. 3.

|  | **Weight of lateral daughters (kg, *W*_A_=*W*_C_)** | | | | | | | | | | |
| --- | --- | --- | --- | --- | --- | --- | --- | --- | --- | --- | --- |
| ***θ*_A_ (degrees, =*θ*_C_)** | **0** | **1** | **2** | **3** | **4** | **5** | **6** | **7** | **8** | **9** | **10** |
| **0** | 0.97 | 1.04 | 1.13 | 1.21 | 1.27 | 1.32 | 1.35 | 1.37 | 1.38 | 1.39 | 1.39 |
| **10** | 0.97 | 1.05 | 1.13 | 1.21 | 1.28 | 1.32 | 1.36 | 1.38 | 1.39 | 1.40 | 1.40 |
| **20** | 0.97 | 1.05 | 1.14 | 1.22 | 1.29 | 1.34 | 1.37 | 1.40 | 1.42 | 1.43 | 1.43 |
| **30** | 0.97 | 1.05 | 1.14 | 1.23 | 1.30 | 1.36 | 1.40 | 1.43 | 1.46 | 1.47 | 1.48 |
| **40** | 0.97 | 1.05 | 1.15 | 1.24 | 1.32 | 1.39 | 1.45 | 1.49 | 1.52 | 1.54 | 1.56 |
| **50** | 0.97 | 1.05 | 1.15 | 1.26 | 1.35 | 1.43 | 1.50 | 1.56 | 1.60 | 1.64 | 1.66 |
| **60** | 0.97 | 1.05 | 1.16 | 1.28 | 1.39 | 1.49 | 1.57 | 1.65 | 1.72 | 1.77 | 1.81 |
| **70** | 0.97 | 1.05 | 1.17 | 1.30 | 1.43 | 1.55 | 1.67 | 1.77 | 1.87 | 1.95 | 2.03 |
| **80** | 0.97 | 1.06 | 1.18 | 1.32 | 1.47 | 1.63 | 1.78 | 1.93 | 2.08 | 2.21 | 2.34 |
| **90** | 0.97 | 1.06 | 1.19 | 1.35 | 1.53 | 1.72 | 1.92 | 2.14 | 2.36 | 2.60 | 2.84 |
