# Supplementary material for: Tree Branching: Leonardo da Vinci's Rule versus Biomechanical Models
Source: PLoS One. 2014 Apr 8;9(4):e93535. doi: 10.1371/journal.pone.0093535 (PMC3979699; doi:10.1371/journal.pone.0093535)
Supplement: Table S5 — Numerical data of Fig. 4B . (DOC) [file pone.0093535.s005.doc]

Table S5. Numerical data of Fig. 4B.

|  | **Weight of daughter A (kg)** | | | | | | | | | | |
| --- | --- | --- | --- | --- | --- | --- | --- | --- | --- | --- | --- |
| ***θ*_A_ (degrees, =*θ*_B_)** | **0** | **1** | **2** | **3** | **4** | **5** | **6** | **7** | **8** | **9** | **10** |
| **0** | 0.99 | 1.13 | 1.20 | 1.26 | 1.29 | 1.32 | 1.34 | 1.36 | 1.38 | 1.39 | 1.40 |
| **10** | 1.00 | 1.14 | 1.21 | 1.27 | 1.30 | 1.33 | 1.35 | 1.37 | 1.39 | 1.40 | 1.41 |
| **20** | 1.02 | 1.17 | 1.24 | 1.29 | 1.33 | 1.36 | 1.39 | 1.40 | 1.42 | 1.43 | 1.44 |
| **30** | 1.06 | 1.22 | 1.29 | 1.35 | 1.39 | 1.42 | 1.44 | 1.46 | 1.48 | 1.49 | 1.50 |
| **40** | 1.13 | 1.29 | 1.38 | 1.43 | 1.48 | 1.51 | 1.53 | 1.55 | 1.57 | 1.58 | 1.60 |
| **50** | 1.23 | 1.41 | 1.50 | 1.56 | 1.61 | 1.65 | 1.67 | 1.70 | 1.71 | 1.73 | 1.74 |
| **60** | 1.39 | 1.60 | 1.70 | 1.77 | 1.82 | 1.86 | 1.90 | 1.92 | 1.94 | 1.96 | 1.97 |
| **70** | 1.68 | 1.92 | 2.05 | 2.13 | 2.20 | 2.25 | 2.29 | 2.32 | 2.34 | 2.36 | 2.38 |
| **80** | 2.34 | 2.68 | 2.85 | 2.97 | 3.06 | 3.13 | 3.18 | 3.22 | 3.26 | 3.29 | 3.31 |
| **90** | 13.13 | 2.75 | 15.56 | 16.17 | 16.67 | 3.70 | 17.45 | 17.77 | 18.06 | 18.33 | 18.57 |
