# Supplementary material for: Tree Branching: Leonardo da Vinci's Rule versus Biomechanical Models
Source: PLoS One. 2014 Apr 8;9(4):e93535. doi: 10.1371/journal.pone.0093535 (PMC3979699; doi:10.1371/journal.pone.0093535)
Supplement: Table S6 — Numerical data of Fig. 5 . (DOC) [file pone.0093535.s006.doc]

Table S6. Numerical data of Fig. 5

|  | **Weight of lateral daughters (kg, *W*_A_=*W*_C_)** | | | | | | | | | | |
| --- | --- | --- | --- | --- | --- | --- | --- | --- | --- | --- | --- |
| ***θ*_A_ (degrees, =*θ*_C_)** | **0** | **1** | **2** | **3** | **4** | **5** | **6** | **7** | **8** | **9** | **10** |
| **0** | 0.99 | 1.27 | 1.40 | 1.49 | 1.55 | 1.59 | 1.63 | 1.66 | 1.68 | 1.70 | 1.71 |
| **10** | 0.99 | 1.27 | 1.40 | 1.49 | 1.55 | 1.60 | 1.63 | 1.66 | 1.69 | 1.70 | 1.72 |
| **20** | 0.99 | 1.27 | 1.41 | 1.50 | 1.57 | 1.62 | 1.65 | 1.68 | 1.71 | 1.73 | 1.75 |
| **30** | 0.99 | 1.28 | 1.42 | 1.52 | 1.59 | 1.64 | 1.69 | 1.72 | 1.75 | 1.77 | 1.79 |
| **40** | 0.99 | 1.28 | 1.44 | 1.54 | 1.62 | 1.69 | 1.73 | 1.78 | 1.81 | 1.84 | 1.86 |
| **50** | 0.99 | 1.29 | 1.46 | 1.58 | 1.67 | 1.74 | 1.80 | 1.85 | 1.89 | 1.93 | 1.96 |
| **60** | 0.99 | 1.30 | 1.48 | 1.62 | 1.72 | 1.81 | 1.89 | 1.95 | 2.00 | 2.05 | 2.09 |
| **70** | 0.99 | 1.31 | 1.51 | 1.66 | 1.79 | 1.90 | 2.00 | 2.08 | 2.15 | 2.22 | 2.28 |
| **80** | 0.99 | 1.32 | 1.54 | 1.72 | 1.88 | 2.02 | 2.14 | 2.26 | 2.36 | 2.46 | 2.55 |
| **90** | 0.99 | 1.34 | 1.58 | 1.78 | 1.98 | 2.16 | 2.33 | 2.49 | 2.65 | 2.80 | 2.95 |
